# Complete sequence of the avian pathogenic *Escherichia coli* strain VTB96933v containing a novel conjugative antimicrobial resistance and virulence IncI1-ST114-ColBM plasmid

**DOI:** 10.1128/mra.00284-25

**Published:** 2025-06-12

**Authors:** Zhuohao Wang, David Gerber, Vincent Perreten

**Affiliations:** 1 Division of Molecular Bacterial Epidemiology & Infectious Diseases, Institute of Veterinary Bacteriology, Vetsuisse Faculty, University of Bern 27210, Bern, Switzerland; 2 College of Veterinary Medicine, Nanjing Agricultural University 70578, Nanjing, Jiangsu, China; 3 V-Tech Veterinary Solutions (Pty) Ltd, Midrand, South Africa; 4 Dunevax Biotech (Pty) Ltd, Windhoek, Namibia; Wellesley College, Wellesley, Massachusetts, USA

**Keywords:** Col plasmid, APEC, virulence, antimicrobial resistance

## Abstract

The complete sequence of a South African avian pathogenic *Escherichia coli* was obtained through short and long read consensus-based assembly. It consists of a 5,120,371 bp chromosome and two plasmids, one of 86,932 and the other of 161,786 bp. The latter is a novel conjugative ColBM plasmid type IncI1-ST114, containing antimicrobial resistance and APEC-associated virulence genes.

## ANNOUNCEMENT

The pathogenicity of avian pathogenic *Escherichia coli* (APEC) is associated with several virulence genes, some found on colicin V (ColV) and colicin B and M (ColBM) plasmids, which so far belong to the IncF type (1, 2). During a whole-genome sequence (WGS)-based investigation of APEC in South Africa, strain VTB96933v presented herein was found to harbor a novel ColBM plasmid type containing both virulence and antimicrobial resistance genes (ARGs).

VTB96933v was isolated from the carcass of a broiler with airsacculitis from a large broiler production farm of above 100,000 animals in South Africa in 2021. The air sac lesions were sampled with a sterile cotton swab, which was spread onto chromogenic tryptone bile X-glucuronide agar (AEC-Amersham) and subsequently incubated at 37°C for 24 h. Colonies were streaked on trypticase soy agar II with 5% sheep blood (TSA-S) (Becton Dickinson). The strain was identified as *E. coli* using MALDI-TOF mass spectrometry (Bruker) (3) and cryopreserved.

VTB96933v was sequenced using Illumina and Oxford Nanopore Technologies (ONT). Genomic DNA (gDNA) for Illumina sequencing was extracted from a lawn of colonies cultivated on TSA-S overnight at 37°C, following the Nextera DNA Flex Microbial Colony Extraction protocol (Illumina). Libraries were prepared using Nextera DNA Flex Library Prep Kit (Illumina) and sequenced (2 × 150 bp paired-end reads) on a NovaSeq 6000 system (S4 flow cell, 300 cycles) (NGSP, University of Bern). Illumina reads were quality-controlled (QC) and trimmed using FASTQC version 0.12.1 (4) and Trimmomatic version 0.39, respectively (5). ONT library was obtained from gDNA extracted using Qiagen DNeasy Blood and Tissue Kit using the SQK-NB114-24 ligation kit and sequenced on a FLO-FLG114 R10 flow cell using the MinION Mk1B device (ONT). DNA quality and quantity were assessed by NanoDrop spectrophotometer and Qubit fluorometer, respectively. ONT reads were base-called, demultiplexed using Dorado version 0.5.3 (https://github.com/nanoporetech/dorado), and QC by Nanoplot version 1.40.2 (6). Illumina and ONT reads were assembled using Trycycler version 0.5.4 (7) and Medaka version 1.11.3 (https://github.com/nanoporetech/medaka) and polished using Polypolish version 0.6.0 (8) to obtain circular plasmids and chromosomes rotated to DnaA. Assemblies were QC using QUAST version 5.2.0 (9) and annotated using PGAP version 6.8 (10). Molecular serotyping and sequence typing were performed using SeqSphere+ version 9.0 (Ridom). Average nucleotide identity (97.09%) to *E. coli* type strain ATCC 11775 (GCA_003697165.1) was calculated using OrthoANIu (11). Virulence and ARGs were detected using ABRicate version 1.0.0 (https://github.com/tseemann/abricate) with VFDB (12) and ResFinder version 4.6.0 (13) databases. Insertion sequences were identified using ISfinder (https://isfinder.biotoul.fr/) (14). Default parameters were used for all software. WGS statistics and characteristics are listed in Table 1.

**TABLE 1:** Sequence statistics and genomic characteristics of avian pathogenic *E. coli* strain VTB96933v from a broiler with airsacculitis in South Africa^*a*^^,*b*^

Parameters	Genomic features
Genome	Size in bp (GenBank acc. no.)
Chromosome	5,120,371 (CP178659)
Plasmids	
pVTB933v-1	161,786 (CP178657)
pVTB933v-2	86,932 (CP178658)
GC content (%)	50.5
Illumina statistics	
Read length	151
Number of paired reads (total)	3,812,717 (7,625,434)
Mean depth	213×
ONT statistics	
*N*_50_ (bp)	12,071
Mean read length (bp)	8,192.9
Number of reads	33,815
Coverage	51.6×
Total number of	
CDSs	5,093
rRNAs	22
tRNAs	88
ncRNAs	11
ANI (%)	97.09
Antibiotic resistance genes	
Chromosome	–
Plasmids	
pVTB933v-1	*tet*(A); *aph(3')-Ia*
pVTB933v-2	*bla*_TEM-1B_; *aac (3)-IId*
Virulence genes	
Chromosome	*shuV*; *chuASTUWXY*; *gspCDEFGHIJKLM*; *kpsMT*; *neuABCDE*; *kpsCDEFU*; *iucABCD*; *iutA*; *rpoS*; *rcsB*; *gndA*; *fyuA*/*psn*; *ybtETU*; *irp1*; *irp2*; *ybtAPQSX*; *espL1*; *espR1*; *vgrG*/*tssI*; *phoP*; *csgABCDEFG*; *ompA*; *espX2*; *fur*; *entABCE*; *fepABCDEG*; *entDFS*; *fes*; *ibeB*; *allB*; *acrB*; *espY3*; *fdeC*; *ykgK*/*ecpR*; *yagVWXYZ*/*ecpABCDE*; *rhs*/*PAAR*; *vgrG*/*tssI*; *hcp2*/*tssD2*; *tssBCFGJKL*; *clpV*/*tssH*; *tssAM*; *hcp1*/*tssD1*; *espY2*; *espY1*; *espX1*; *fimABCDEFGH*; *pmrA*; *espX5*; *espX4*; *espL4*; *ibeC*; *aslA*; *espY5*
Plasmids	
pVTB933-1	*iroBCDEN*
pVTB933-2	–

^*a*^ CDSs, coding DNA sequences; rRNAs, ribosomal RNAs; tRNAs, transfer RNAs; ncRNAs, non-coding RNAs; and ANI, average nucleotide identity.

^*b*^ “–” indicates absence.

**Fig 1:**
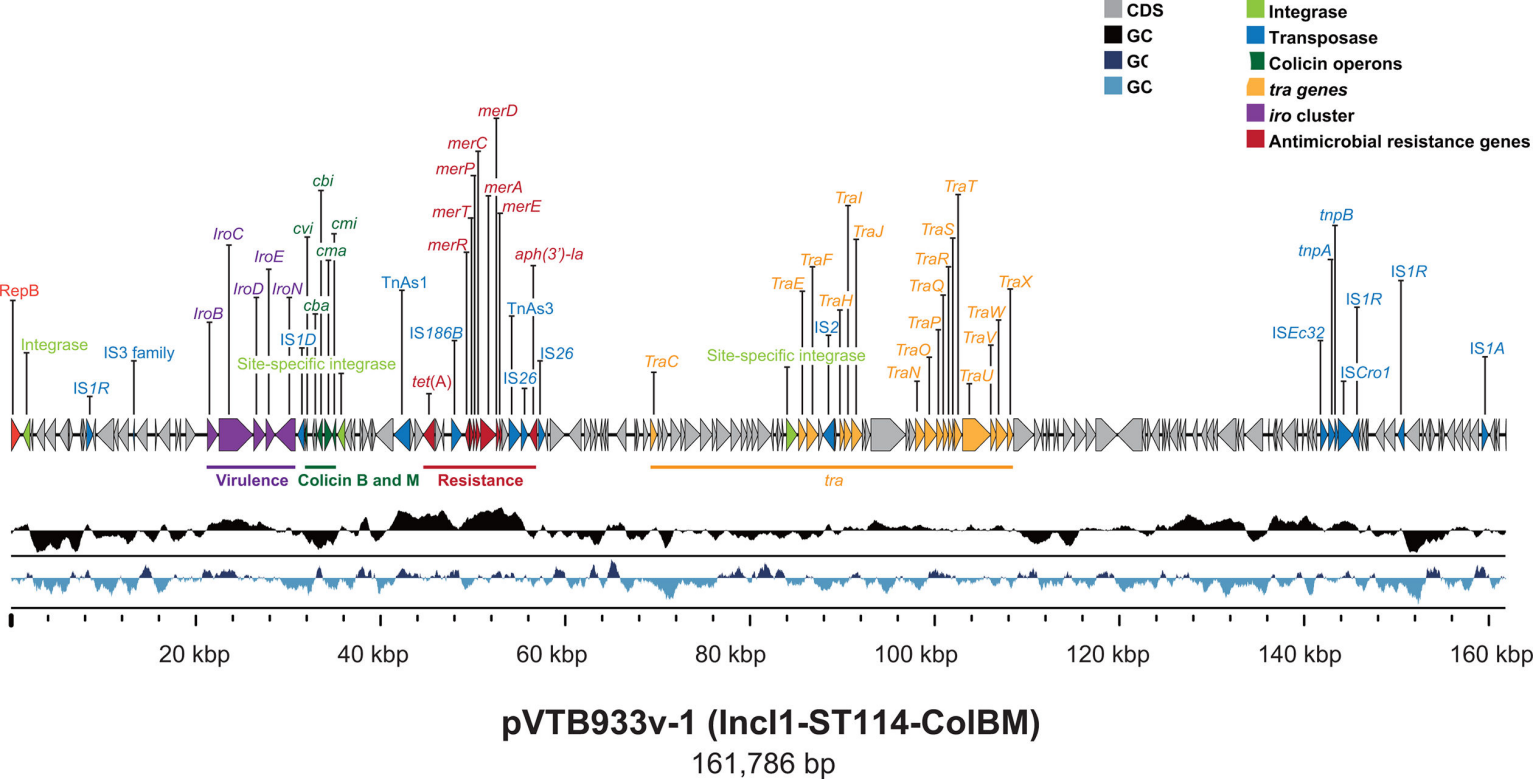
Genetic map of the 161,786 bp antimicrobial resistance and virulence IncI1-ST114-ColBM plasmid pVTB933v-1 of the avian pathogenic *E. coli* strain VTB96933v from a broiler with airsacculitis in South Africa. The map includes the size of the plasmid in base pairs, GC content and distribution, and GC skew. Coding sequences (CDSs) are indicated with arrows, including those for the virulence *iro* cluster in purple, antimicrobial resistance genes in red, *tra* genes in orange, and colicin operons in green. Virulence and antimicrobial resistance genes and functions: *tet*(A), tetracycline resistance efflux; *aph(3')-Ia*, aminoglycoside phosphotransferase for resistance to kanamycin, neomycin, paromomycin, ribostamycin, and lividomycin; *merACDEPRT*, mercury resistance operons; *iroBCDEN*: salmochelin siderophore virulence system. Transposase and integrase are indicated in blue and light green, respectively. The map was plotted using Clinker version 0.0.28 (19) and adapted with Adobe Illustrator version 29.3.1.

VTB96933v (serogroup O86:H2; ST349) contained one circular chromosome and two circular plasmids (pVTB933v-1 and pVTB933v-2), which belonged to IncI1-ST114-ColBM and IncF (F16:A-:B-), respectively, as determined by plasmid PubMLST (15). In addition to belonging to IncI1, pVTB933v-1 exhibited a unique structure as determined by an NCBI BLASTN search (16). It contained APEC-associated salmochelin siderophore virulence genes (17), ARGs for tetracycline, aminoglycosides, mercury, transfer-associated genes, and ColBM operons (Fig. 1). Conjugal transfer of pVTB933v-1 occurred into *E. coli* J53dR (18) (10^−6^ transconjugants/donor) after selection on 30 µg/mL kanamycin and was confirmed by ONT sequencing as above. Additional ARGs and virulence genes were identified on pVTB933v-2 and the chromosome, respectively (Table 1).

## Data Availability

Complete sequences of VTB96933v, pVTB933v-1, and pVTB933v-2 have been deposited into GenBank under accession numbers CP178659, CP178657, and CP178658. The raw reads of VTB96933v were deposited into the Sequence Read Archive (SRA) under accession numbers SRX24732945 (Illumina) and SRX27934570 (ONT), BioProject number PRJNA1117783, and BioSample number SAMN41579897.
